# Prevalence, attitudes and concerns toward telepsychiatry and mobile health self-management tools among patients with mental disorders during and after the COVID-19 pandemic: a nationwide survey in Poland from 2020 to 2023

**DOI:** 10.3389/fpsyt.2023.1322695

**Published:** 2024-01-08

**Authors:** Monika Dominiak, Adam Gędek, Anna Z. Antosik, Paweł Mierzejewski

**Affiliations:** ^1^Department of Pharmacology, Institute of Psychiatry and Neurology, Warsaw, Poland; ^2^Department of Psychiatry, Faculty of Medicine, Collegium Medicum, Cardinal Wyszynski University in Warsaw, Warsaw, Poland

**Keywords:** mHealth, mobile health, telehealth, digital health, smartphone, APP, acceptance, expectation

## Abstract

**Introduction:**

Mobile Health (mHealth) is a rapidly growing field of medicine that has the potential to significantly change everyday clinical practice, including in psychiatry. The COVID-19 pandemic and technological developments have accelerated the adoption of telepsychiatry and mobile solutions, but patient perceptions and expectations of mHealth remain a key factor in its implementation.

**Aim:**

The aim of this study was to assess (1) the prevalence, (2) attitudes, preferences and (3) concerns about mobile mental health, including telepsychiatry and self-management tools, among patients with mental disorders over the period 2020–2023, i.e., at the onset, peak and after the expiration of the COVID-19 pandemic.

**Materials and methods:**

A semi-structured survey was administrated to 354 patients with mental disorders in Poland. The questions were categorized into three section, addressing prevalence, attitudes, and concerns about telepsychiatry and mobile health self-management tools. The survey was conducted continuously from May 2020 to the end of May 2023.

**Result:**

As many as 95.7% of patients with mental disorders used mobile devices at least once a week. Over the course of 3 years (from 2020 to 2023), there was a significant increase in the readiness of patients to embrace new technologies, with the percentage rising from 20% to 40%. In particular, a remarkable growth in patient preferences for telepsychiatry was observed, with a significant increase from 47% in 2020 to a substantial 96% in 2023. Similarly, mHealth self-management tools were of high interest to patients. In 2020, 62% of patients like the idea of using mobile apps and other mobile health tools to support the care and treatment process. This percentage also increased during the pandemic, reaching 66% in 2023. At the same time, the percentage of patients who have concerns about using m-health solutions has gradually decreased, reaching 35% and 28% in 2023 for telepsychiatry and for the reliability and safety of m-health self-management tools, respectively.

**Conclusion:**

This study highlights the growing acceptance of modern technologies in psychiatric care, with patients showing increased readiness to use telepsychiatry and mobile health self-management tools, in particular mobile applications, after the COVID-19 pandemic. This was triggered by the pandemic, but continues despite its expiry. In the face of patient readiness, the key issue now is to ensure the safety and efficacy of these tools, along with providing clear guidelines for clinicians. It is also necessary to draw the attention of health systems to the widespread implementation of these technologies to improve the care of patients with mental disorders.

## 1 Introduction

Mobile Health (mHealth) is a rapidly developing field that leverages the capabilities of mobile devices, including smartphones, patient monitoring tools, personal digital assistants (PDAs), and various wireless devices, to enhance healthcare (1). In recent years, the field of mobile health has seen significant growth and change, both in prevalence and the features provided. The widespread availability of the internet and smartphones, offering mobile applications, have been driving this trend. Mobile Health solutions have already been implemented for a few years in the field of psychiatry and first experiences show that they can be a valuable element to complement the coordinated and personalized care. These solutions can support the diagnostics, patient education, therapeutic process, as well as self-management in illness (2). Especially, they can also be very helpful in monitoring chronic, recurrent mental disorders on a continuous, real-time basis (3–7). There is also a great potential for improving communication with specialist (3–7). Remote communication via text message, telephone, video call or chat, known as telemedicine, has been known for a long time. It was a good option for patients with limited access to health care facilities. In the face of the pandemic, most patients confronted the problem of not being able to consult a specialist as before. This has resulted in an explosive demand for remote contact, causing telepsychiatry to develop in a literally revolutionary way. Remote solutions might make it easier to contact health care professionals also during emergencies, health crises and suicide threats.

Smartphones and mobile applications, due to their increasing availability, low price, and simple functionality, can be excellent tools to support people with mental health problems. Nevertheless, a key factor influencing the use of these solutions in psychiatric care is patients perception. The results of studies analyzing the opinions of patients participating in clinical trials using m-health devices are highly inconsistent. Studies conducted among patients with bipolar disorder (BD) and schizophrenia indicated high level of satisfaction and acceptance of the tested interventions (8, 9). Similarly, an app dedicated to preventing self-aggressive behaviors among youth was also highly rated (10). It is worth nothing, however, that these studies focused on patients in a relatively stable mental state and lasted for a short time, up to a few months. Clinical observations, on the other hand, suggest that interest in a given tool decreased with longer use. In one study, the interest in long-term use of wearables with built-in sensors among BD patients was examined. The results revealed that patients were not keen on using these tools over an extended period, even though the same patients reported high satisfaction and acceptance of the tool in a three-month study (11). Walsh et al. conducted a review of patients’ opinions using mHealth tools for monitoring mental states. An analysis of 57 papers showed that patients were generally content with their usage, but the proposed solutions did not entirely meet their expectations (12). Patients pointed out the need for more personalized and flexible approaches that align with their preferences and needs, are user-friendly, and genuinely helpful. Negative aspects of mHealth tools were also highlighted, such as the annoying and stressful continuous reminders of their illness, a feeling of being monitored, loss of dignity, and autonomy (12–14). In a Polish study evaluating the feasibility of monitoring the mental state of patients with BD and predicting mood phase changes using a dedicated mobile application (ChADMon), a high dropout rate (44%) was observed at one-year follow-up (15). However, to date, no study has assessed attitudes, concerns, and expectations toward mHealth solutions in psychiatry in the Polish population.

The studies cited are from before the pandemic. The COVID-19 pandemic has served as a significant stimulus for the development of mental mobile healthcare, due to disruption of conventional doctor-patient interactions. In particular, over the course of pandemic, there was a notable growth in the adoption of telepsychiatry in comparison to previous years (16, 17). This trend correlated with concomitant mental deterioration and increased psychiatric symptoms in the population during the pandemic (18–20). The pandemic certainly forced a rapid transformation of contact with specialist through remote system. Therefore, it is interesting to explore how this form of contact was evaluated by patients during the peak of the pandemic, and how this is shaping up after the pandemic expires and the possibility of returning to the traditional form of contact. Due to the difficulty in contacting a specialist, many patients had to cope on their own during the most critical period. Regardless of telepsychiatry, there was a rapid growth of the mobile app market during this period as an attempt to respond to increased medical needs. However, it has not been studied how the pandemic period affected the use of and attitudes toward the tools offered by mobile health. In particular, it is interesting to explore how this has affected two of the most prominent areas of mHealth in psychiatry, namely telepsychiatry and mHealth self-management tools for mental health support. Therefore we decided to explore how perceptions among patients of these solutions were shaped at the beginning of the pandemic—where change was forced, through its peak, to its extinction and the associated possibility of a return to traditional forms of treatment. This can provide valuable information on the further development of mHealth in psychiatry.

The purpose of this study was to assess the prevalence, attitudes, preferences and concerns about telepsychiatry and mHealth self-management tools, among patients with mental disorders over the period 2020–2023, i.e., at the onset, peak and after the expiration of the COVID-19 pandemic. The specific aims of this study were: (1) to assess the prevalence and usage of mobile devices and mHealth solutions and evaluate changes in this field between 2020 and 2023; (2) to assess attitudes, expectations, and preferences toward telepsychiatry and mHealth self-management tools in psychiatry, and to evaluate changes in this field between 2020 and 2023; (3) to assess concerns and risks associated with telepsychiatry and mHealth self-management tools in psychiatry and evaluate changes in this field between 2020 and 2023.

## 2 Materials and methods

A semi-structured, a 28-item questionnaire was developed (see Supplementary material), taking into consideration existing literature, insights into specific aspects of mental disorders, and knowledge from clinicians at the Institute of Psychiatry and Neurology. These clinicians had prior experience in conducting Polish research involving mHealth app in the field of psychiatry. The questions aimed to explore the characteristics of mHealth solutions, including telepsychiatry and mobile apps that would be acceptable for long-term use from a patients’ perspective. We sought to assess patients’ needs, expectations, and potential applications of mobile technologies in managing their mental health.

At the forefront of the questionnaires, there is brief information about the purpose of the survey. This is followed by research questions, both closed and open, covering the following three main areas:

1. the prevalence of mobile device and internet usage and patients’ current experiences with the use of mHealth solutions in the area of health and mental health (what percentage of respondents already have some experience, how they evaluate the tested solutions);
2. patients’ attitudes, opinions, and preferences regarding mHealth solutions in psychiatry, in particular views regarding telepsychiatry and self-management tools. The questions relate to interest in the use of mobile technology in psychiatry, the opportunities that it can offer, the level of readiness to use it, factors influencing patients’ attitudes (including physician/psychologist influence), needs, expectations and areas of application of mHealth technology from the patient’s perspective, preferences for the selection of specific self-management tools, and preferences for specific functions of mobile apps;
3. concerns and risks associated with the adoption of mHealth solutions for mental health management.

Additionally, the questionnaire concluded with clinical and demographic data collection. Data is collected on age, gender, place of residence, education, occupational status. The questionnaire also includes a section on the respondent’s health (a checkbox for the type of mental disorder the respondent suffers from) and a Patient Global Impression (PGI) scale, on which the respondent assesses his or her general health on a scale of 1–7 points.

The online survey was administered to individuals seeking mental health support who willingly participated in the study. Distribution involved reaching out to individuals receiving care within mental health facilities and counseling centers across 16 regions (voivodeships) in Poland. The distribution process spanned from May 2020 to the end of May 2023 on a continuous basis, resulting in the participation of 354 patients, representing a response rate of 84%. The specific inclusion criteria for participants were as follows: (1) diagnosis of any mental disorder; (2) residence in Poland; (3) age 18 or older; (4) consent to participate in the survey.

The study adhered to ethical guidelines and was communicated to the Bioethics Committee at the Institute of Psychiatry and Neurology in Warsaw, Poland. Formal approval from the Bioethics Committee was deemed unnecessary since the survey posed no threat to the participants’ well-being and interests. Data were handled with utmost confidentiality, fairness, and equality, in accordance with the Helsinki principles.

To meet the survey’s aim 1, we conducted a summative analysis of responses regarding the prevalence and usage of mobile devices and mHealth solutions from 2020 to 2023, and then compared the obtained responses between the years 2020–2023 using the Chi-square test. If the difference was statistically significant, we conducted additional comparisons for each year considering the Bonferroni correction. To address aim 2 of the study, we conducted a summative analysis of responses regarding the attitudes, expectations, and preferences toward telepsychiatry and mHealth self-management tools in psychiatry and then compared the obtained responses between the years 2020–2023 using the Chi-square test for nominal-scale variables. If the difference was statistically significant, we conducted additional comparisons for each year considering the Bonferroni correction. Kruskal-Wallis ANOVA was used for ordinal-scale variables with non-normal distributions. In addition, a qualitative analysis of open-ended questions was conducted. To address aim 3 of the study, we conducted a summative analysis of responses regarding the concerns and risks associated with the telepsychiatry and mHealth self-management tools in psychiatry and then compared the obtained responses between the years 2020–2023 using the Chi-square test. If the difference was statistically significant, we conducted additional comparisons for each year considering the Bonferroni correction. In addition, a qualitative analysis of open-ended questions regarding patients’ concerns was conducted.

Statistical analysis was conducted using Statistica 13.3 software. Descriptive statistics included means, standard deviations, medians, and interquartile ranges for data that did not meet normal distribution criteria. Chi-square tests were applied for nominal-scale variables, while Kruskal-Wallis ANOVA was used for ordinal-scale variables with non-normal distributions. In instances of small group sizes, the chi-square test aggregated lower-ranked responses, ensuring result interpretability. For post-hoc comparisons, the Bonferroni correction was employed to enhance the test’s reliability. Relationships between variables were assessed through regression and correlation methods, exploring the impact of demographic factors on the responses. Statistical significance was determined at *p* < 0.05.

## 3 Results

### 3.1 General characteristics

A total of 354 respondents completed the online survey (*n* = 354). All closed questions were completed by respondents (100%). The general characteristics of patients are presented in Table 1. The information about the health of respondents is presented in Table 2.

**Table 1:** General characteristics of the respondents (*n* = 354).

Variables		Year (*n* = 354)			
		2020 (*n* = 78)	2021 (*n* = 70)	2022 (*n* = 88)	2023 (*n* = 118)
Sex	Female	60.3% (*n* = 47)	60% (*n* = 42)	63.6% (*n* = 56)	54.2% (*n* = 64)
	Male	39.7% (*n* = 31)	40% (*n* = 28)	36.4% (*n* = 32)	45.8% (*n* = 54)
Age	18–24	10.2% (*n* = 8)	5.7% (*n* = 4)	2.3% (*n* = 2)	5.9% (*n* = 7)
	25–39	37.2% (*n* = 29)	22.9% (*n* = 16)	34.1% (*n* = 30)	32.2% (*n* = 38)
	40–55	33.3% (*n* = 26)	42.8% (*n* = 30)	47.7% (*n* = 42)	47.5% (*n* = 56)
	55–64	16.7% (*n* = 13)	20% (*n* = 14)	12.5% (*n* = 11)	11.9% (*n* = 14)
	>65	2.6% (*n* = 2)	8.6% (n = 6)	3.4% (*n* = 3)	2.5% (*n* = 3)
Education	Lower secondary	16.7% (*n* = 13)	22.9% (*n* = 16)	23.9% (*n* = 21)	28.8% (*n* = 34)
	Professional	14.1 (*n* = 11)	21.4% (*n* = 15)	17% (*n* = 15)	10.2% (*n* = 12)
	Higher	69.2% (*n* = 54)	55.7% (*n* = 39)	59.1% (*n* = 52)	61% (*n* = 72)
Professional activity	Student	11.5% (*n* = 9)	4.3% (*n* = 3)	2.3% (*n* = 2)	4.2% (*n* = 5)
	Working	82.1% (*n* = 64)	71.4% (*n* = 50)	71.6% (*n* = 63)	89.8% (*n* = 106)
	Pensioner	6.4% (*n* = 5)	15.7% (*n* = 11)	6.8% (*n* = 6)	5.1% (*n* = 6)
	Other	0% (*n* = 0)	8.6% (*n* = 6)	19.3% (*n* = 17)	0.9% (*n* = 1)
Residence	City > 250,000	53.9% (*n* = 42)	44.3% (*n* = 31)	46.6% (*n* = 41)	56% (*n* = 66)
	City 50,000–250,000	17.9% (*n* = 14)	24.3% (*n* = 17)	27.3% (*n* = 24)	22% (*n* = 26)
	City < 50,000	20.5% (*n* = 16)	17.1% (*n* = 12)	20.4% (*n* = 18)	17.8 (*n* = 21)
	Village	7.7% (*n* = 6)	14.3% (*n* = 10)	5.7% (*n* = 5)	4.2% (*n* = 5)

**Table 2:** Health information of the respondents (*n* = 354).

Variables		Percent (number)
“I am treated for…”	Depression	63% (*n* = 223)
	Anxiety	20.3% (*n* = 72)
	Sleep disorders	14.4% (*n* = 51)
	Addictions	12.4% (*n* = 44)
	Bipolar disorder	10.2% (*n* = 36)
	Neurosis	2% (*n* = 7)
	Schizophrenia	1.4% (*n* = 5)
	Other	2.8% (*n* = 10)
“At present, I feel…”	Very seriously ill	0.3% (*n* = 1)
	Seriously ill	1.1% (*n* = 4)
	Noticeably ill	7.1% (*n* = 25)
	Moderately ill	33.6% (*n* = 119)
	Slightly ill	26.3% (*n* = 93)
	Almost healthy	27.1% (*n* = 96)
	Healthy	4.5% (*n* = 16)

### 3.2 Prevalence and usage of mobile devices and mHealth solutions—results for aim 1

#### 3.2.1 Prevalence and usage of mobile devices and mHealth solutions—aggregate analysis (2020–2023)

**Figure 1:**
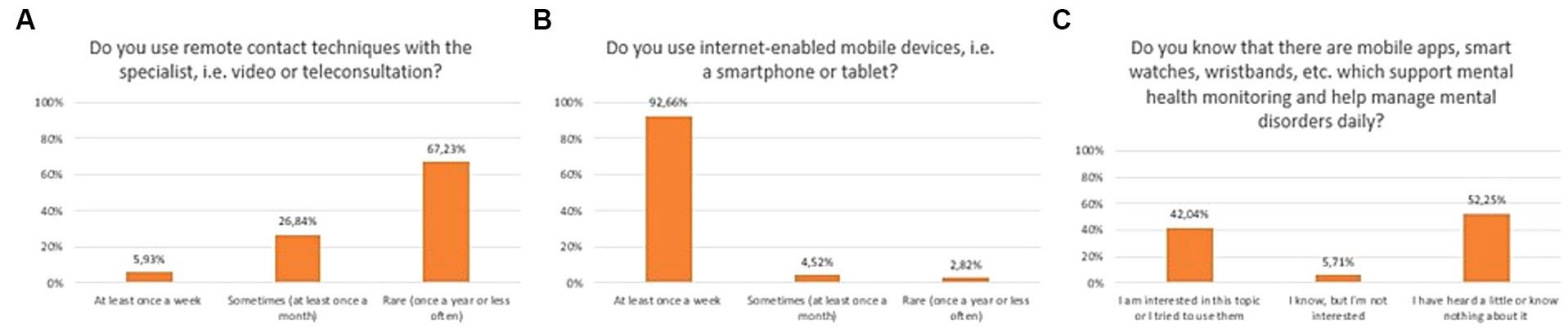
Responses regarding prevalence and usage of mobile devices and mHealth solutions among mental health patients; **(A)** Usage of remote techniques to communicate with specialists; **(B)** Usage of mobile devices; **(C)** Awareness of mHealth tools dedicated to mental health patients.

A significant number of respondents declared that they use mobile devices at least once a week (92.7%). Thirty-two percent use remote contact with a specialist frequently (once a week to once a month), while 67.2% use it only once a year or less often. Forty-two percent of patients stated that they have heard about mHealth tools (such as apps, smart watches, wristbands etc.), or already used them. Simultaneously, 52.3% of respondents had heard little or nothing about it. The exact distribution of responses to questions related to the prevalence and usage of new technologies is presented in Figure 1.

#### 3.2.2 Changes in prevalence and usage of mobile devices and mHealth solutions between 2020 and 2023

Prevalence of using remote contact techniques with mental health specialist between 2020 and 2023 did not change statistically significantly (Chi^2^ = 7,621,773, df = 3, *p* = 0.05451). Similarly, awareness of mHealth tools in psychiatry between 2020 and 2023 did not change statistically significantly (Chi^2^ = 6,731,831, df = 3, *p* = 0.08095).

### 3.3 Attitudes, expectations, and preferences toward telepsychiatry and mHealth self-management tools in psychiatry—results for aim 2

#### 3.3.1 Attitudes, expectations, and preferences toward telepsychiatry and mHealth self-management tools in psychiatry—aggregate analysis (2020–2023)

**Figure 2:**
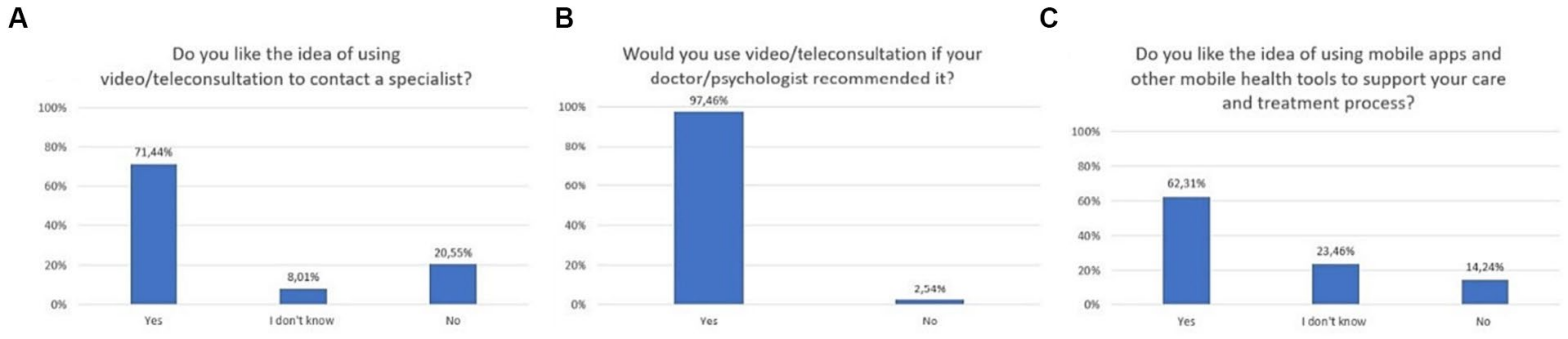
Responses regarding attitudes, expectations, and preferences; **(A)** Attitudes toward video/teleconsultation; **(B)** Readiness to use video/teleconsultation upon recommendation by a doctor/psychologist; **(C)** Attitudes toward mHealth tools.

**Figure 3:**
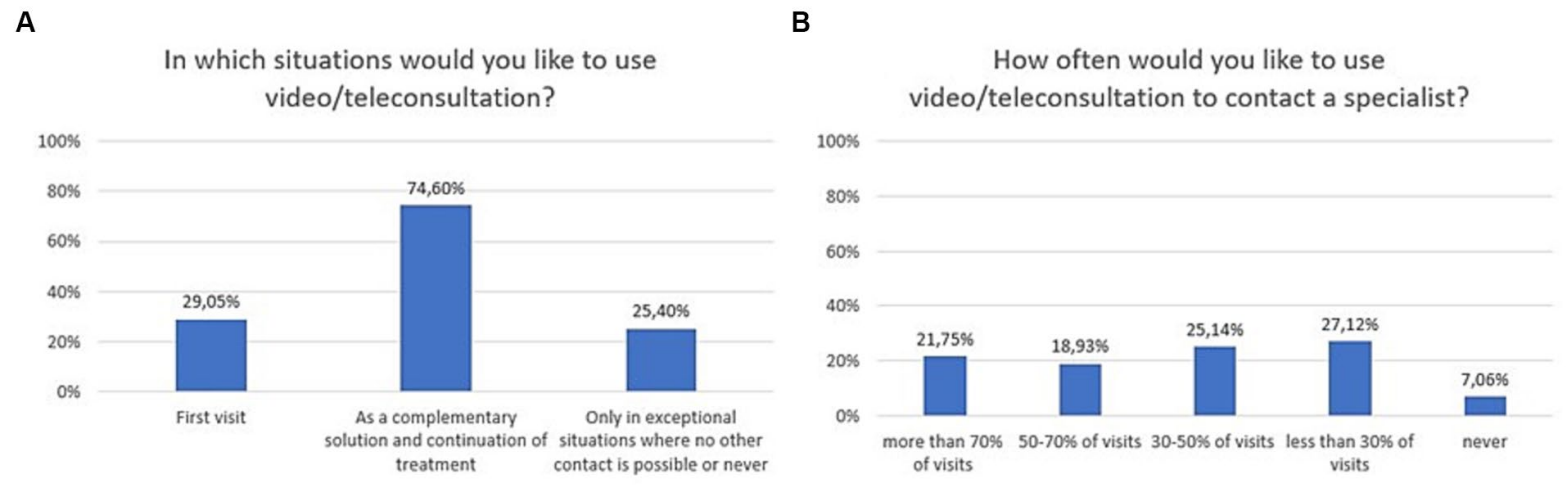
Responses regarding attitudes, expectations, and preferences; **(A)** Situations in which respondents would like to use video/teleconsultations (multiple-choice question); **(B)** Expected frequency of video/teleconsultation.

The majority of respondents liked the idea of using video/teleconsultation to contact a specialist (71.4%), and as much as 97.5% would use them if that was the recommendation of the specialist. Sixty-two percent of respondents liked the idea of using mobile apps and other mobile health tools to support care and treatment process (Figure 2). In a multiple-choice question, the majority of respondents declared that telepsychiatry could be applied as a complementary tool, used to continue treatment (74.6%). According to 29.1%, remote contact could be applicable from the first visit (Figure 3). As much as 40.7% of respondents stated that they would like video/teleconsultations to account for more than 50% of all visits. 96.6% of respondents would use a mental health mobile app if that was the recommendation of the specialist. The exact distribution of responses to main questions related to attitudes toward video/teleconsultations is shown in Figures 2, 3.

The great majority of respondents declared that mental health mobile apps could be useful for patients through the following features: educational (95.8%), self-motivating (93.8%), self-monitoring of well-being with visualization of this date (94.9%), monitoring activity to detect early signs of deterioration (78.8%), therapy support—relaxation module (94.7%), therapy support—medications reminders (94.7%), therapy support—allowing on-going communication with the doctor/psychologist (95.5%). Responses to an open-ended question about other useful features did not contain any indications other than the above.

#### 3.3.2 Changes in attitudes, expectations, and preferences toward telepsychiatry and mHealth self-management tools between 2020 and 2023

**Figure 4:**
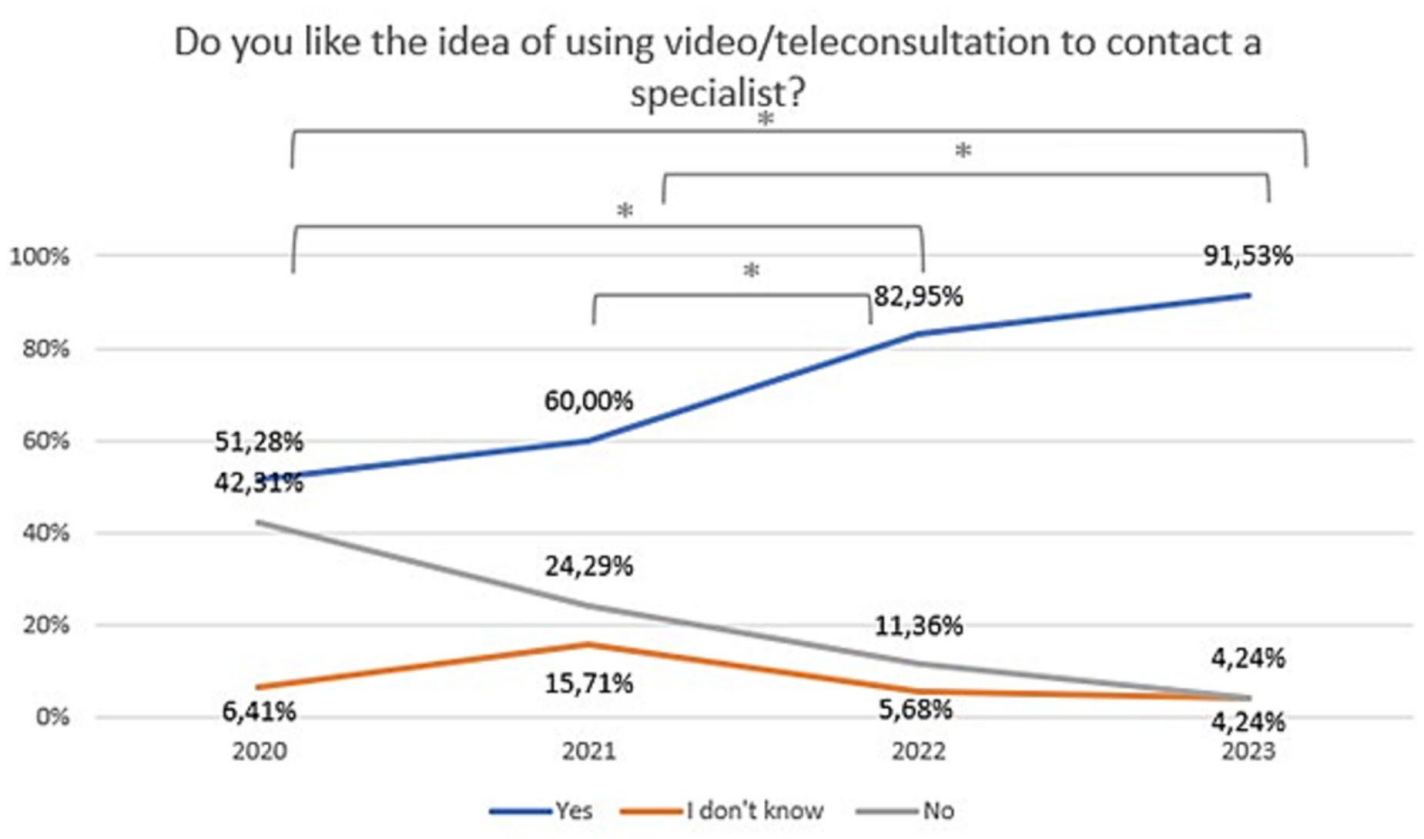
Responses regarding idea of using video/teleconsultation over the period 2020–2023. ^*^Statistically significant difference.

**Figure 5:**
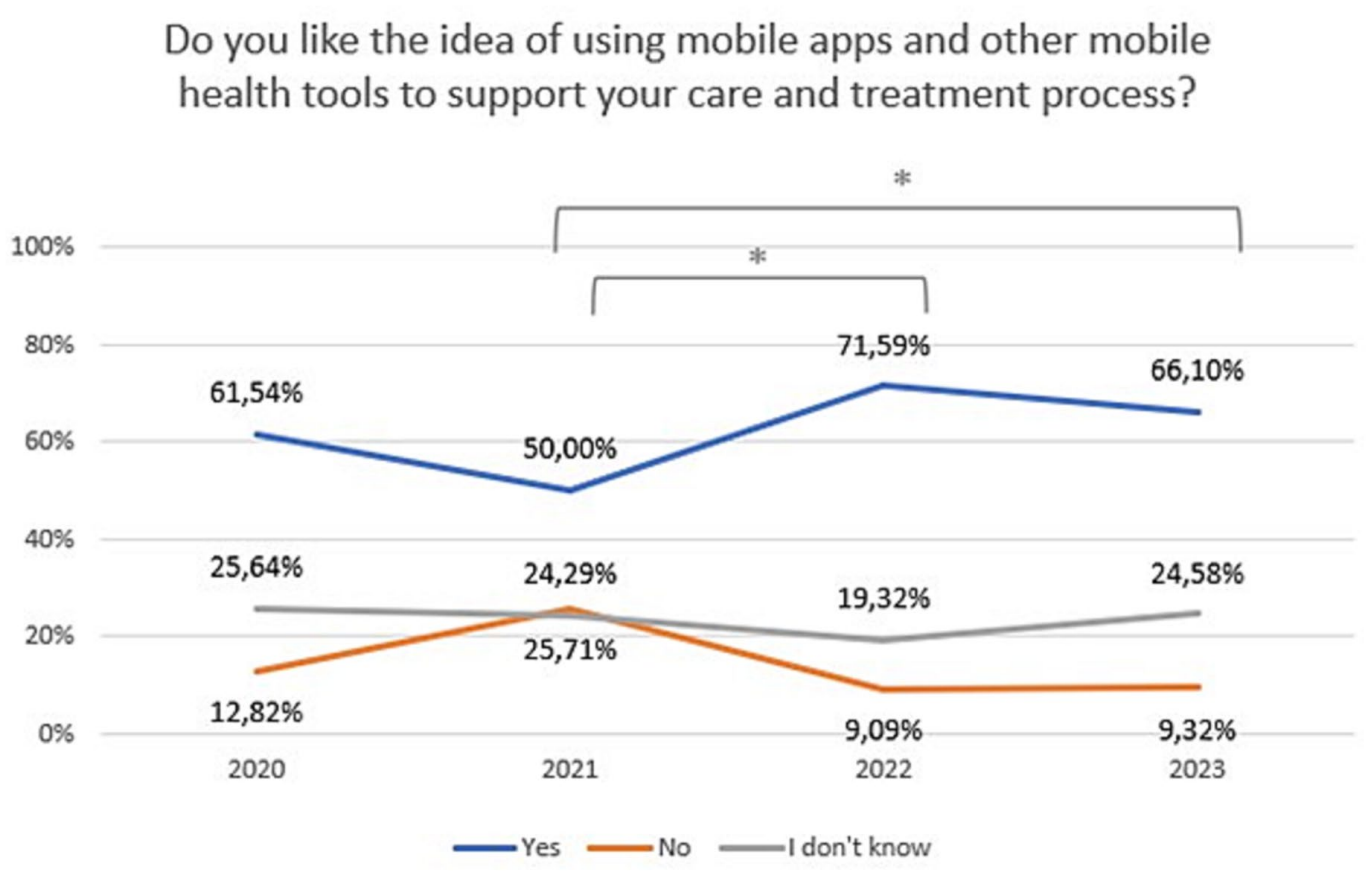
Responses regarding idea of using mobile technology over the period 2020–2023. ^*^Statistically significant difference.

**Figure 6:**
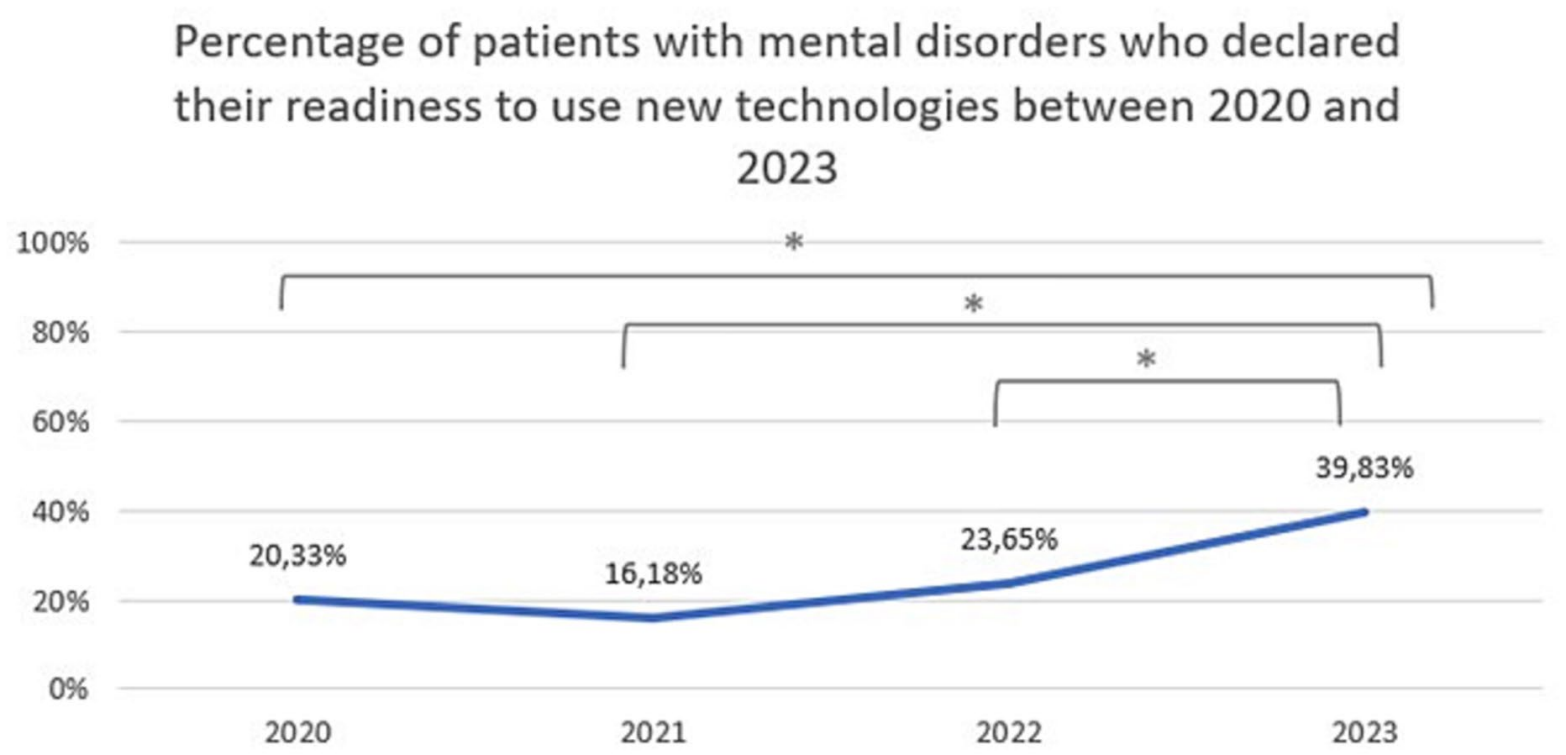
The percentage of patients with mental disorders who declared they were ready to use new technologies between 2020 and 2023. ^*^Statistically significant difference.

A statistically significant difference was found between 2020 and 2023 regarding attitudes toward video/teleconsultation (Chi^2^ = 62,60,504, df = 6, *p* = 0.00000). The Bonferroni correction was included in the comparisons for each year (statistically significance: 2020 vs. 2022: Chi^2^ = 21,41,480, df = 2, *p* = 0.00002; 2020 vs. 2023: Chi^2^ = 45,61,123, df = 2, *p* = 0.00000; 2021 vs. 2022: Chi^2^ = 10,50,707, df = 2, *p* = 0.00523; 2021 vs. 2023:Chi^2^ = 27,36,393, df = 2, *p* = 0.00000). The trend is presented in Figure 4. A statistically significant difference was also found between 2020 and 2023 regarding attitudes toward mHealth tools (Chi^2^ = 14,70,202, df = 6, *p* = 0.02271). The Bonferroni correction was included in the comparisons for each year (statistically significance: 2021 vs. 2022: Chi^2^ = 9,924,326, df = 2, *p* = 0.00700; 2021 vs. 2023: Chi^2^ = 9,550,167, df = 2, *p* = 0.00844). The trend is presented in Figure 5. The percentage of mental health patients, who declared they were ready to use new technologies between 2020 and 2023 is presented in Figure 6 (Chi^2^ = 15,93,151, df = 3, *p* = 0.00117; statistically significance: 2020 vs. 2023: Chi^2^ = 8,381,067, df = 1, *p* = 0.00379; 2021 vs. 2023: Chi^2^ = 14,26,987, df = 1, *p* = 0.00016; 2022 vs. 2023: Chi^2^ = 7,254,401, df = 1, *p* = 0.00707; Figure 6).

**Figure 7:**
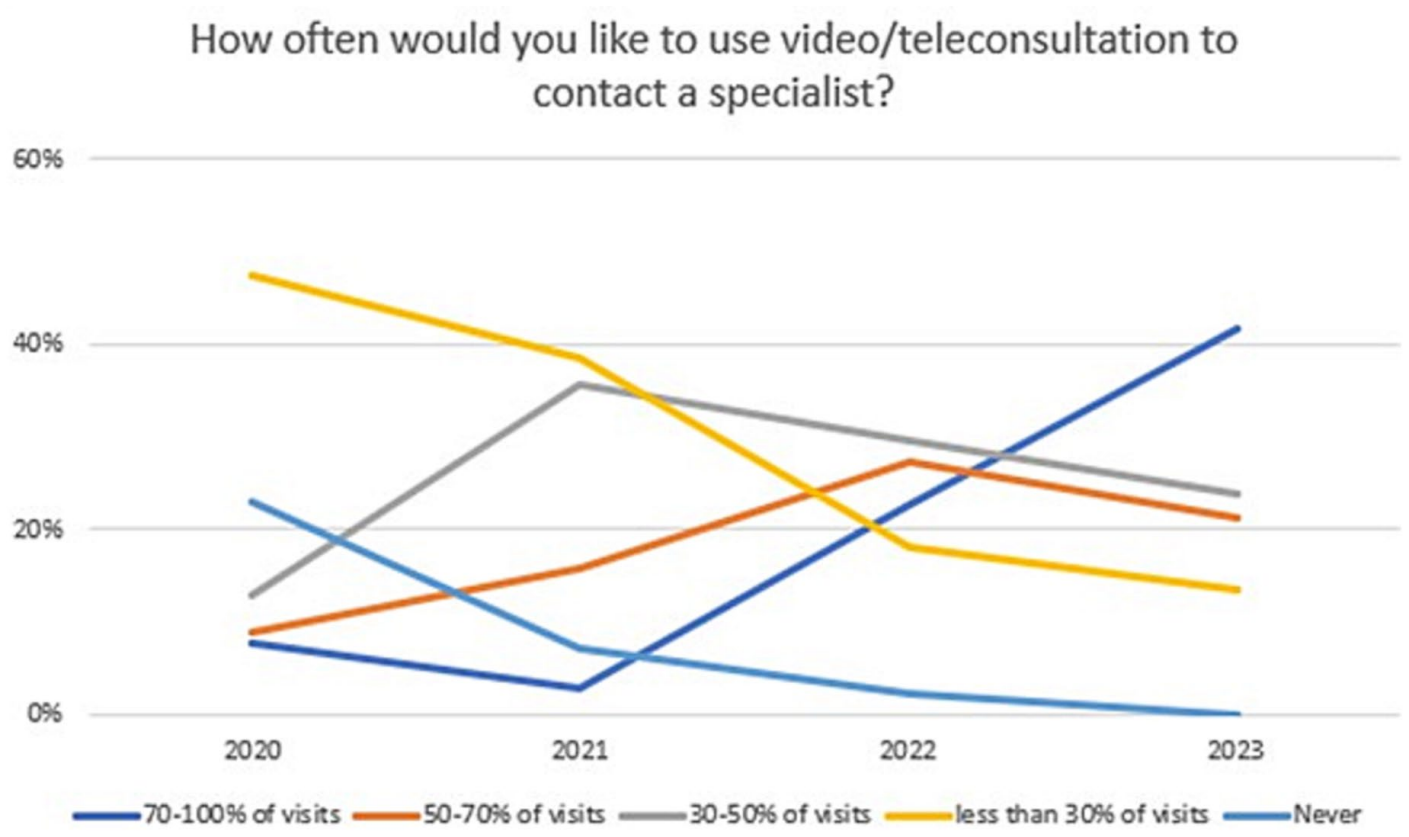
Responses regarding the declared desire for frequency of use video/teleconsultation over the period 2020–2023.

**Figure 8:**
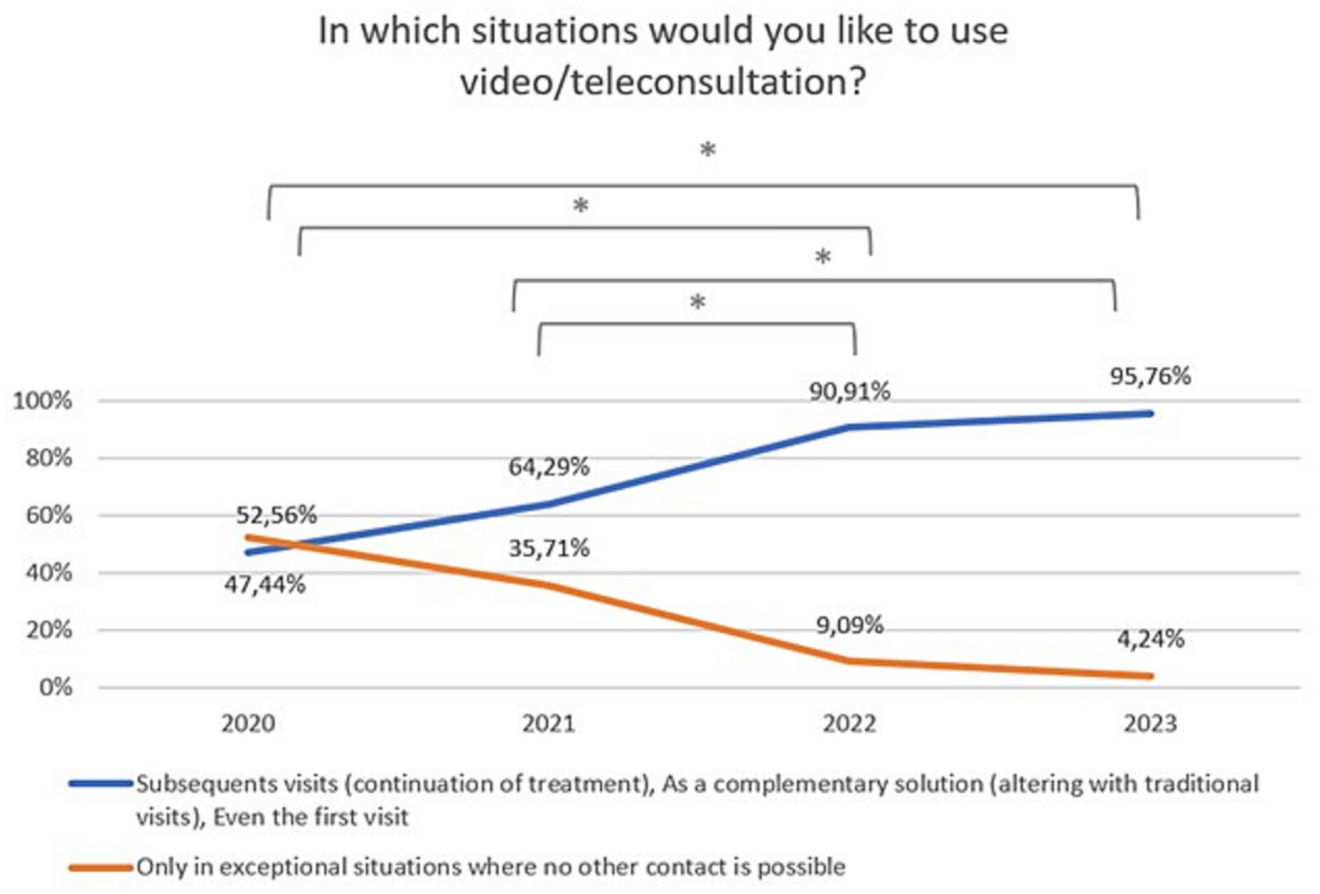
Responses regarding the desire to use video/teleconsultation in specific situations over the period 2020–2023. ^*^Statistically significant difference.

Changes in the declared intention to use video/teleconsultation frequency are shown in Figure 7. The variable was tested on an ordinal scale and the Kruskalla-Wallis ANOVA test showed statistical significance (H = 95.12151, *p* = 0.000). Over the period 2020–2023, there was an increase in the preference for the use of video/teleconsultation from 47.4% in 2020 to 95.8% in 2023. Relevant responses were combined to ensure that the size of the test group was appropriate (Chi^2^ = 79,53,916, df = 3, *p* = 0.00000; statistically significance: 2020 vs. 2022: Chi^2^ = 37,56,181, df = 1, *p* = 0.00000; 2020 vs. 2023: Chi^2^ = 61,06044, df = 1, *p* = 0.00000; 2021 vs. 2022: Chi^2^ = 16,72,400, df = 1, *p* = 0.00004; 2021 vs. 2023: Chi^2^ = 32,45,982, df = 1, *p* = 0.00000; Figure 8).

#### 3.3.3 Attitudes, expectations and preferences toward telepsychiatry and mHealth self-management tools—analysis of open-ended questions (2020–2023)

In response to why patients like the idea of video/teleconsultation as a tool to contact a specialist, the majority mentioned to convenience and simplicity of such a solution (*n* = 38). In addition, respondents pointed to time-saving (*n* = 36), the only possible form of contact during the pandemic (*n* = 47), the feeling of security (*n* = 56), and fast contact (*n* = 32). One person each indicated the possibility of constant contact, access to better specialists, and the assurance of anonymity in small towns. Among the respondents who did not like this idea, they mentioned that it will not replace live contact (*n* = 18), that they prefer traditional contact (*n* = 29), and they fear being recorded/overheard (*n* = 48). One person each indicated the difficulty of establishing a relationship, the uncomfortable feeling, the inability to talk quietly at home, and that it is convenient for doctors, not patients.

The majority of respondents who answered the question what they would like to improve indicated the quality and stability of the connection (*n* = 52), the ability to connect by video, as talking alone is insufficient (*n* = 18), security and privacy (*n* = 55), more personalized solutions (*n* = 45). One person each pointed to the possibility of remanding by SMS, compatibility on various devices, and ability to send files.

Patients gave a variety of responses as to why they liked the idea of using mobile technology, such as additional help in managing disease daily (*n* = 43), opportunity for additional interaction with the health service/medical doctor (*n* = 53), convenience (*n* = 56), mobilization and reminder (*n* = 31), assistance in the treatment process (*n* = 43). Among those who did not like this idea, respondents mentioned that they have no experience with such solutions (*n* = 18), see them as entertainment, or gadgets (*n* = 12), are afraid of privacy (*n* = 52), and would not be able to use them (*n* = 5).

### 3.4 Concerns and risks toward telepsychiatry and mHealth self-management tools in psychiatry—results for aim 3

#### 3.4.1 Concerns and risks associated with the use of telepsychiatry and mHealth self-management tools in psychiatry—aggregate analysis (2020–2023)

**Figure 9:**
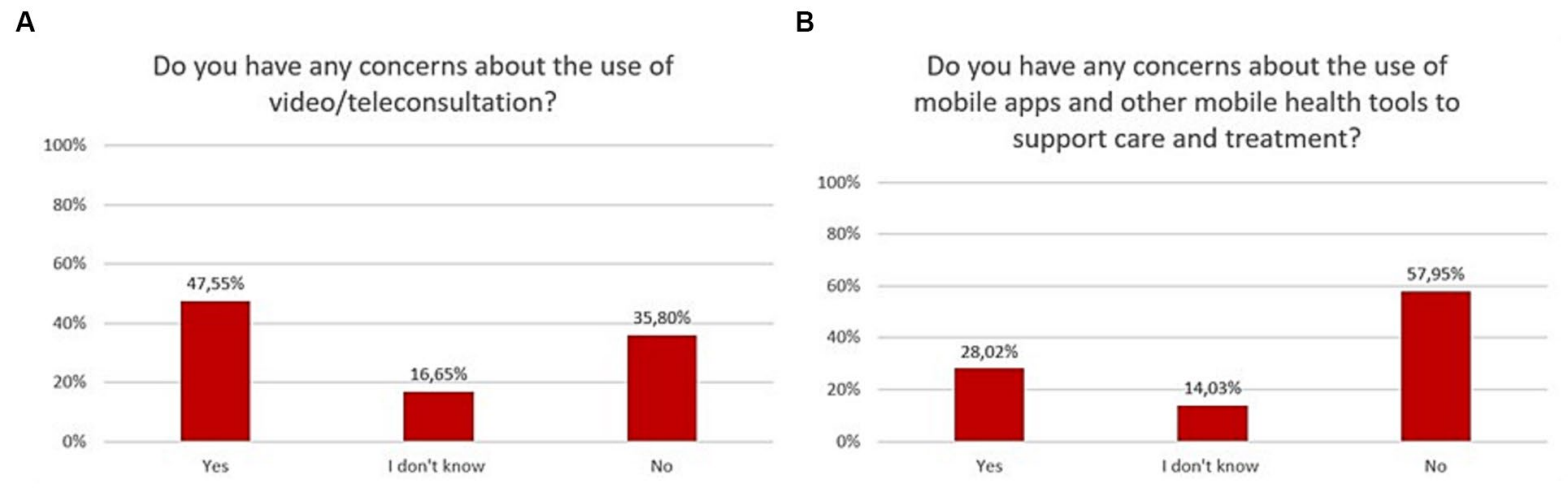
Responses regarding concerns and risks associated with the use of mHealth in psychiatry; **(A)** video/teleconsultation concerns; **(B)** mHealth tools concerns.

Slightly less than half of respondents (47.6%) had some concerns about the use of video/teleconsultation, while only 28% had concerns about the use of mobile apps and other mHealth tools to support care and treatment. The exact distribution of responses to questions related to concerns over the use of new technologies is shown in Figure 9.

#### 3.4.2 Change in perceived concerns and risks associated with the use of telepsychiatry and mHealth self-management tools in psychiatry between 2020 and 2023

**Figure 10:**
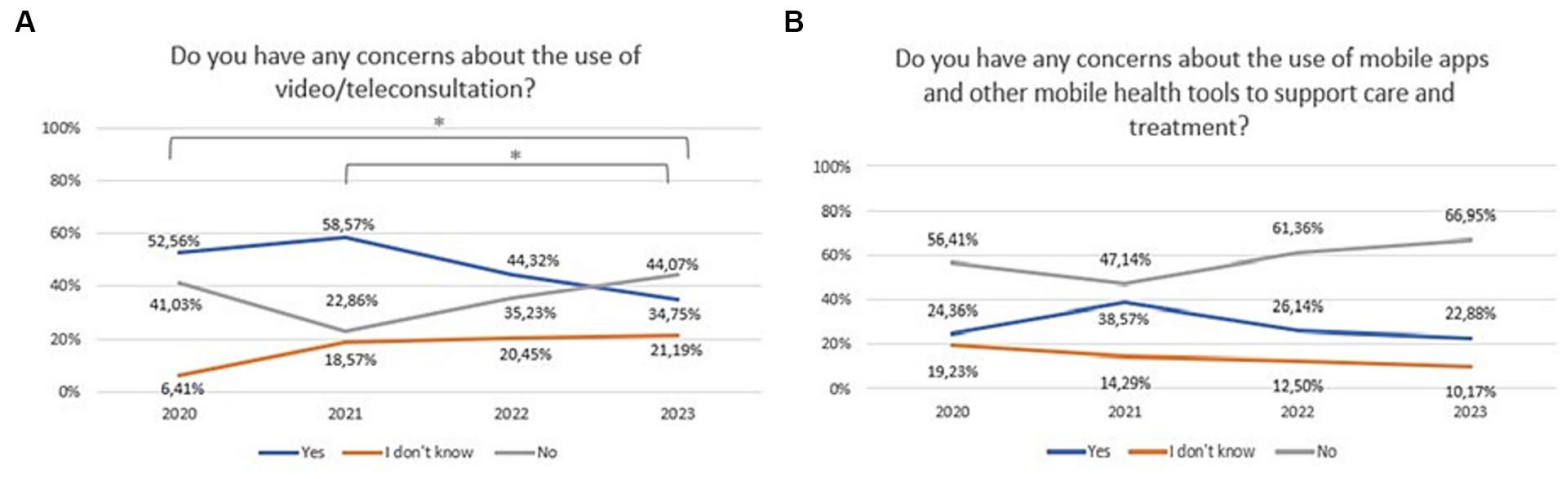
Responses regarding concerns about: **(A)** the use of video/teleconsultation over the period 2020–2023; **(B)** the use of mobile apps and other mobile health tools to support care and treatment over the period 2020–2023. ^*^Statistically significant difference.

Concerns about video/teleconsultation in psychiatry between 2020 and 2023 are shown in Figure 10 (Chi^2^ = 19,23,567, df = 6, *p* = 0.00378). The Bonferroni correction was included in the comparisons for each year (statistically significance: 2020 vs. 2023: Chi^2^ = 10,36,361, df = 2, *p* = 0.00562; 2021 vs. 2023: Chi^2^ = 11,33,167, df = 2, *p* = 0.00346). Concerns toward mHealth tools in psychiatry between 2020 and 2023 are shown in Figure 10 (Chi^2^ = 10,44,849, df = 6, *p* = 0.10699).

#### 3.4.3 Concerns and risks associated with the use of telepsychiatry and mHealth self-management tools-analysis of open-ended questions (2020–2023)

Among the concerns about video/teleconsultations in the open questions, respondents mentioned: privacy and security (*n* = 56), lack of comfort in conversation (*n* = 31), inability to talk to a medical doctor live (*n* = 14), superficial visits (*n* = 32), medical mistakes (*n* = 4), devices support (*n* = 2).

Among the concerns about mobile tools in the open questions, respondents mentioned: privacy and security (*n* = 51), uncertainty about the effectiveness of these tools (*n* = 31), lack of reliable data on clinical usefulness (*n* = 20), clinicians’ hesitancy to recommend these tools (*n* = 14), and low-tech level (*n* = 8), lack of experience with a reliable solution (*n* = 4). One patient pointed out that such solutions may harm patients (*n* = 1).

### 3.5 Influence of age, sex, education, professional activity, place of residence, and health status on readiness to use new technologies

The Kruskal-Wallis test showed no statistically significant differences in readiness to use new technologies between groups depending on age (H = 4; *p* = 0.24), education (H = 2; *p* = 0.53), professional activity (H = 6, *p* = 0.43), place of residence (H = 3, *p* = 0.05), and health status (H = 6; *p* = 0.14). The U-Mann–Whitney test showed no statistically significant differences in readiness to use new technologies between groups depending on sex (U = 14,217,00, *p* = 0.32).

## 4 Discussion

The challenges, that the 21st century has brought, include mental health problems, which are now major contributors to the global burden of disease (21). Unfortunately, most people in need of psychiatric treatment are not getting the help. Above all, high costs and a shortage of clinicians stand in the way. Negative social attitudes toward psychiatric treatment and perceived stigma may also contribute. It is estimated that the treatment gap for people with mental disorders is as high as 50% on average worldwide (22). In countries with high income, this percentage is obviously lower, but the limitation in accessibility is still present due to other factors. Common barriers to seeking treatment include lack of time, transport issues or availability of professionals in the patient’s area. However, the 21st century has also brought new options for dealing with mental disorders. Mobile health has begun to play a role in modern psychiatric care. The health system is increasingly embracing new digital solutions such as telepsychiatry. This helps to make mental health care more accessible, but also opens up entirely new possibilities, such as mobile applications to support diagnosis, monitoring and treatment. Some of these solutions are already in place and some, such as telepsychiatry, have become routine clinical practice since the COVID-19 pandemic. This implementation, however, is taking place at different rates in countries worldwide, often at suboptimal levels (23). This is also the case in Poland. No significant impact has yet been made on mainstream mental health care, despite the many solutions that are available. One obstacle here may be the targeted users’ knowledge of and attitudes toward mobile health solutions. Therefore, in this study we aimed at assessing the prevalence, attitudes and concerns regarding new technologies in mental health care, specifically, about telepsychiatry and mHealth self-management tools, among individuals with mental disorders.

The first optimistic finding is that significant number of respondents declared that they use mobile devices at least once a week (93%). This already shows that the vast majority of people with mental disorders own and use mobile devices with internet access. Second, that readiness to use new technologies among patients increased from 20% in 2020 to 40% in 2023. Moreover, their willingness to use new technological solutions in psychiatry has also increased significantly over the last 3 years, and currently one in two to three patients would like to be supported in this way. In particular, there was a significant increase in the preference for the use of telepsychiatry (video or teleconsultation) from 47% in 2020 to as much as 96% in 2023. Importantly, 97.5% would have used them if they had been recommended by a specialist. However, in terms of personal experience, on average only 32% of patients with mental disorders used telepsychiatry quite often (between once a week and once a month) between 2020 and 2023. This may indicate that the needs and expectations of patients are greater than the provision of these services. Telepsychiatry undoubtedly has many benefits that are perceived by patients with mental disorders. It certainly increases patients’ access to professional psychiatric help by making them independent of their location and mobility. In addition, patients may feel more comfortable during an online visits in the comfort of their own home than in a face-to-face meeting. Other advantages pointed out by patients in our survey were as follows: simplicity of such solutions, time-saving, or fast and constant contact with specialists. Concluding, with the emergence of the pandemic, the last 3 years have been a revolution in the development of telepsychiatry. Patients, though initially coerced, are now mostly convinced of its advantages. In 2023, although the pandemic has subsided, the preference for using remote contact with a specialist persists.

Given that such services are as effective as traditional visits in treating depression and improving quality of life (24), the creation of a well-organized system of telepsychiatry appears to be an opportunity for many previously untreated patients. This also applies to the therapeutic process. A recent systematic review showed that video-consultation-based psychotherapy is as effective as traditional visits in reducing symptoms of depression (25). However, patients also had some concerns about telepsychiatry. These were expressed by as many as 53% of patients in 2020, but this percentage dropped significantly to 35% in 2023. These include fear of being overheard or recorded, concerns about losing contact with a known model such as face-to-face consultation, the problem of establishing a relationship, or the issues of confidentiality and privacy during the connection.

As regards the mHealth self-management tools (such as mobile apps, smartwatches and wristbands), the level of interest and willingness to use was similar to that of telepsychiatry. More than 60% of patients with mental disorders liked the idea of using mobile apps and other mobile health tools to support their care and treatment process. This percentage also increased during the pandemic, reaching 66% in 2023. As to personal experiences, 42% of patients stated that they have heard about self-management tools (such as apps, smart watches, wristbands, etc.), or already used them.

In general, these findings are consistent with those currently available in the literature (26, 27). Patients with mental disorders are ready, open and willing to use the solutions offered by new technologies. Therefore, this factor does not seem to be an obstacle to their wider use. However, 28% of patients expressed some concerns about the use of mHealth tools, it is worth quoting them here, as they seem very well founded and decide on their broader use. Respondents indicated that have concerns about the reliability of these tools and the security of their confidential data. They are also uncertain about the effectiveness of these tools and do not have a clear recommendation from their specialist.

It seems that, the concern is above all about mobile applications that do not meet sufficient clinical validation standards. In the face of the COVID-19 pandemic, there was a surge in the number of apps based on artificial intelligence, marketed as mental health support tools. However, they often failed to meet their stated objectives and even posed real risks to the user (28–30). However, this has laid the foundations for their growing use in supporting mental health. Although there are now thousands of English-language apps related to mental health, only the Headspace and Calm app have any clinical studies indicating effectiveness and safety. At the same time, they rank highest in terms of number of downloads and user activity (31). Despite the mixed findings, for such apps, recommendations tend toward recommending them. Given the low cost and potential accessibility to a wide range of people who would otherwise not have access to support and treatment.

When it comes to implementing a regulatory framework aimed at health apps and their market access, Germany has taken a pioneering role. DiGA (Digitale Gesundheitsanwendungen, in German meaning digital health apps) (32). If an app can prove both compliance with the general requirements and positive clinical effects, the German Federal Agency for Medicines and Health Products approves it and it is reimbursed by insurance. Similar models have been developed in France and Belgium (33). Other European countries, including Poland, have so far chosen approaches with legal obligations and compliance rules based on the General Data Protection Regulation or the Medical Devices Regulation (34, 35).

However, there appears to be a need for specific guidelines in particular countries. Collaboration with World Psychiatric Associations (WPA) member societies will also contribute to the development of evidence-based guidelines for the safe and ethical use of digital mental health tools in countries around the world. This will allow to use the potential of mHealth in the best way and give a broad range of people access to cheap, effective and safe tools to help improve their mental health.

The number of positive attitudes toward telepsychiatry and mHealth self-management tools increased between 2020 and 2023, as we noted earlier. However, one of the interesting findings of our study was that no differences regarding attitudes were found between 2020 and 2021 when COVID-19’s effects were the most severe. Several factors could have contributed to this. In the first place, the effects of the pandemic varied by region over time, thereby affecting the overall statistical significance. Second, the COVID-19 pandemic also had some delayed effects, among which appear to be improved attitudes toward telepsychiatry and mHealth tools. In addition, other external factors, such as the popularity of mobile solutions on an annual basis, are also contributing to this rise.

The results of this survey show that it is not patient attitudes that stand in the way in the wider use of mHealth solutions. With all certainty, it is worth taking advantage of the readiness of people with mental disorders to use mHealth tools. However, one cannot forget about the issues of verifying the effectiveness of these tools and their security, including the security of sensitive data. The lack of clear recommendations for specific tools can also be a problem for clinicians who are not sure which solutions to recommend to patients (28). This may be another reason for the low implementation of new technologies in psychiatry in Poland and worldwide.

This study has certain limitations, that should be acknowledged. Firstly, respondents that chose to participate in the study might be predisposed to a positive attitudes toward telepsychiatry and mHealth and thereby introduce a positive bias. However, the response rate was high which suggest a reliable sample. Secondly, it cannot be assumed that the respondents’ declared interest in mHealth will translate into actual use. Further efforts are needed to help implement these solutions. Thirdly, most data assessed were from patients living in urban areas. Finally, cross-sectional design and self-reported data were also limitations of this study. Therefore, the results should be generalized with particular care.

## 5 Conclusion

In this study, we explored the prevalence, attitudes, preferences and concerns about mobile health, including telepsychiatry and self-management tools among patients with mental disorders over the pandemic period from 2020 to 2023. The survey revealed that patients with mental disorders are increasingly embracing mobile technologies, such as telepsychiatry and self-management tools (mainly mobile applications) to support their treatment. The vast majority (up to 95% of patients with mental disorders) use mobile devices at least once a week. Over the 3 years of the pandemic (from 2020 to 2023), the proportion of patients willing to use mobile solutions increased. In particular, there was a remarkable increase in preference among patients for using telepsychiatry—in 2023 the percentage of respondents choosing this form of contact with a specialist was as high as 96%. Similarly, mHealth self-management tools were of high interest to patients. More than 60% of patients like the idea of using mobile apps and other mobile health tools to support the care and treatment process. This percentage also increased during the pandemic, reaching 66% in 2023. At the same time, the percentage of patients who have concerns about using mHealth solutions decreased. However, in 2023, 35% and 28% of patients still have concerns about telepsychiatry and the reliability and safety of m-health self-management tools, respectively.

Summarizing the results of the survey, there is a clear trend of increasing acceptance of mHealth solutions, along with increasing patient readiness to utilize them. This was triggered by the pandemic, but continues despite its expiry.

The results suggest that there is considerable potential to improve the accessibility and effectiveness of psychiatric care using mHealth technologies. However, caution must be exercised regarding the safety and effectiveness of these tools. This requires, among other things, guidelines for clinicians to recommend safe and effective solutions to patients. In order to meet the growing readiness and expectations of patients and to contribute to the improvement of psychiatric care, the implementation of mHealth solutions in Poland and worldwide requires the urgent attention of health system decision-makers.

## Data availability statement

The raw data supporting the conclusions of this article will be made available by the authors, without undue reservation.

## Author contributions

MD: Conceptualization, Data curation, Formal analysis, Funding acquisition, Project administration, Writing – original draft, Writing – review & editing. AG: Formal analysis, Visualization, Writing – original draft, Writing – review & editing. AA: Funding acquisition, Supervision, Writing – review & editing. PM: Conceptualization, Project administration, Supervision, Writing – review & editing.

## References

[1] World Health Organization. mHealth: new horizons for health through mobile technologies. Geneva, Switzerland: World Health Organization (2011).

[2] Lecomte T Potvin S Corbière M Guay S Samson C Cloutier B. Mobile apps for mental health issues: Meta-review of Meta-analyses. JMIR Mhealth Uhealth. (2020) 8:e17458. doi: 10.2196/17458, PMID: 32348289 PMC7293054

[3] Hidalgo-Mazzei D Mateu A Reinares M Undurraga J Bonnin CD Sanchez-Moreno J. Self-monitoring and psychoeducation in bipolar patients with a smart-phone application (SIMPLe) project: design, development and studies protocols. BMC Psychiatry. (2015) 15:52. doi: 10.1186/s12888-015-0437-6, PMID: 25884824 PMC4379950

[4] Matthews M Abdullah S Murnane E Voida S Choudhury T Gay G. Development and evaluation of a smartphone-based measure of social rhythms for bipolar disorder. Assessment. (2016) 23:472–83. doi: 10.1177/1073191116656794, PMID: 27358214 PMC6155452

[5] Torous J Larsen ME Depp C Cosco TD Barnett I Nock MK. Smartphones, sensors, and machine learning to advance real-time prediction and interventions for suicide prevention: a review of current Progress and next steps. Curr Psychiatry Rep. (2018) 20:51. doi: 10.1007/s11920-018-0914-y, PMID: 29956120

[6] Faurholt-Jepsen M Frost M Ritz C Christensen EM Jacoby AS Mikkelsen RL. Daily electronic self-monitoring in bipolar disorder using smartphones—the MONARCA I trial: a randomized, placebo-controlled, single-blind, parallel group trial. Psychol Med. (2015) 45:2691–704. doi: 10.1017/S0033291715000410, PMID: 26220802

[7] Faurholt-Jepsen M Vinberg M Frost M Debel S Christensen EM Bardram JE. Behavioral activities collected through smartphones and the association with illness activity in bipolar disorder. Int J Methods Psychiatr Res. (2016) 25:309–23. doi: 10.1002/mpr.1502, PMID: 27038019 PMC6860202

[8] Ben-Zeev D Brenner CJ Begale M Duffecy J Mohr DC Mueser KT. Feasibility, acceptability, and preliminary efficacy of a smartphone intervention for schizophrenia. Schizophr Bull. (2014) 40:1244–53. doi: 10.1093/schbul/sbu033, PMID: 24609454 PMC4193714

[9] Faurholt-Jepsen M Busk J Frost M Vinberg M Christensen EM Winther O. Voice analysis as an objective state marker in bipolar disorder. Transl Psychiatry. (2016) 6:e856. doi: 10.1038/tp.2016.123, PMID: 27434490 PMC5545710

[10] Grist R Cliffe B Denne M Croker A Stallard P. An online survey of young adolescent girls’ use of the internet and smartphone apps for mental health support. Bjpsych Open. (2018) 4:302–6. doi: 10.1192/bjo.2018.43 30083383 PMC6066993

[11] Javelot H Spadazzi A Weiner L Garcia S Gentili C Kosel M. Telemonitoring with respect to mood disorders and information and communication technologies: overview and presentation of the PSYCHE project. Biomed Res Int. (2014) 2014:104658. doi: 10.1155/2014/104658, PMID: 25050321 PMC4094725

[12] Walsh S Golden E Priebe S. Systematic review of patients’ participation in and experiences of technology-based monitoring of mental health symptoms in the community. BMJ Open. (2016) 6:e008362. doi: 10.1136/bmjopen-2015-008362, PMID: 27329437 PMC4916567

[13] Saunders KEA Bilderbeck AC Panchal P Atkinson LZ Geddes JR Goodwin GM. Experiences of remote mood and activity monitoring in bipolar disorder: a qualitative study. Eur Psychiatry. (2017) 41:115–21. doi: 10.1016/j.eurpsy.2016.11.005, PMID: 28135594 PMC5947817

[14] Daus H Kislicyn N Heuer S Backenstrass M. Disease management apps and technical assistance systems for bipolar disorder: investigating the patients’ point of view. J Affect Disord. (2018) 229:351–7. doi: 10.1016/j.jad.2017.12.059, PMID: 29331693

[15] Antosik-Wójcinska A Chojnacka M Dominiak M Swiecicki Ł. The use of smartphones in the management of bipolar disorder-mobile apps and voice analysis in monitoring of mental state and phase change detection. Eur Neuropsychopharmacol. (2019) 29:S528–9. doi: 10.1016/j.euroneuro.2018.11.784

[16] Molfenter T Roget N Chaple M Behlman S Cody O Hartzler B. Use of telehealth in substance use disorder services during and after COVID-19: online survey study. JMIR Ment Heal. (2021) 8:e25835. doi: 10.2196/25835, PMID: 33481760 PMC7895293

[17] Molfenter T Heitkamp T Murphy AA Tapscott S Behlman S Cody OJ. Use of telehealth in mental health (MH) services during and after COVID-19. Community Ment Health J. (2021) 57:1244–51. doi: 10.1007/s10597-021-00861-2, PMID: 34165695 PMC8222700

[18] Goodyear T Richardson C Aziz B Slemon A Gadermann A Daly Z. Mental distress and virtual mental health resource use amid the COVID-19 pandemic: findings from a cross-sectional study in Canada. Digit Heal. (2023) 9:205520762311735. doi: 10.1177/20552076231173528, PMID: 37163172 PMC10164262

[19] Hossain MM Tasnim S Sultana A Faizah F Mazumder H Zou L. Epidemiology of mental health problems in COVID-19: a review. F1000Res. (2020) 9:636. doi: 10.12688/f1000research.24457.1, PMID: 33093946 PMC7549174

[20] Wu T Jia X Shi H Niu J Yin X Xie J. Prevalence of mental health problems during the COVID-19 pandemic: a systematic review and meta-analysis. J Affect Disord. (2021) 281:91–8. doi: 10.1016/j.jad.2020.11.117, PMID: 33310451 PMC7710473

[21] GBD 2019 Mental Disorders Collaborators. Global, regional, and national burden of 12 mental disorders in 204 countries and territories, 1990-2019: a systematic analysis for the global burden of disease study 2019. Lancet Psychiatry. (2022) 9:137–50. doi: 10.1016/S2215-0366(21)00395-3 35026139 PMC8776563

[22] Patel V Maj M Flisher AJ De Silva MJ Koschorke M Prince M. Reducing the treatment gap for mental disorders: a WPA survey. World Psychiatry. (2010) 9:169–76. doi: 10.1002/j.2051-5545.2010.tb00305.x, PMID: 20975864 PMC2953637

[23] Volpe U Ramalho R Orsolini L Ransing R de Filippis R Gürcan A. An update from the WPA working group on digitalization in mental health and care. World Psychiatry. (2023) 22:494–5. doi: 10.1002/wps.21143, PMID: 37713546 PMC10503917

[24] Shih Y-H Wang J-Y Chou P-H Lin K-H. The effects of treatment via telemedicine interventions for patients with depression on depressive symptoms and quality of life: a systematic review and meta-ranalysis. Ann Med. (2023) 55:1092–101. doi: 10.1080/07853890.2023.2187078, PMID: 36920229 PMC10026747

[25] Guaiana G Mastrangelo J Hendrikx S Barbui C. A systematic review of the use of Telepsychiatry in depression. Community Ment Health J. (2021) 57:93–100. doi: 10.1007/s10597-020-00724-2, PMID: 33040191 PMC7547814

[26] Guracho YD Thomas SJ Win KT. Smartphone application use patterns for mental health disorders: a systematic literature review and meta-analysis. Int J Med Inform. (2023) 179:105217. doi: 10.1016/j.ijmedinf.2023.105217 37748330

[27] Gutiérrez-Rojas L Alvarez-Mon MA Andreu-Bernabeu Á Capitán L de Las CC Gómez JC. Telepsychiatry: the future is already present. Rev Psiquiatr Salud Ment. (2022) 16:51–7. doi: 10.1016/j.rpsm.2022.09.001, PMID: 37689522

[28] Cowan KE McKean AJ Gentry MT Hilty DM. Barriers to use of Telepsychiatry: clinicians as gatekeepers. Mayo Clin Proc. (2019) 94:2510–23. doi: 10.1016/j.mayocp.2019.04.018, PMID: 31806104

[29] Heaven W. Hundreds of AI tools have been built to catch covid. None of them helped. MIT Technology Review. (2021) Available at: https://www.technologyreview.com/2021/07/30/1030329/machine-learning-ai-failed-covid-hospital-diagnosis-pandemic (Accessed 1 October 2023)

[30] Golinelli D Boetto E Carullo G Nuzzolese AG Landini MP Fantini MP. Adoption of digital Technologies in Health Care during the COVID-19 pandemic: systematic review of early scientific literature. J Med Internet Res. (2020) 22:e22280. doi: 10.2196/22280, PMID: 33079693 PMC7652596

[31] Lau N O’Daffer A Colt S Yi-Frazier JP Palermo TM McCauley E. Android and iPhone Mobile apps for psychosocial wellness and stress management: systematic search in app stores and literature review. JMIR Mhealth Uhealth. (2020) 8:e17798. doi: 10.2196/17798, PMID: 32357125 PMC7275252

[32] Devices FI for D and M. DiGA directory. (2022) Available at: https://diga.bfarm.de/de/verzeichnis (Accessed 1 October 2023)

[33] MHealthBelgium. Belgian platform for medical mobile applications. (2022) Available at: https://mhealthbelgium.be/ (Accessed 1 October 2023)

[34] E-L. Regulation (EU) 2016/679 of the European Parliament and of the Council of 27 April 2016 on the protection of natural persons with regard to the processing of personal data and on the free movement of such data, and repealing Directive 95/46. (2016) Available at: https://eur-lex.europa.eu/legal-content/EN/TXT/PDF/?uri=CELEX:32016R0679.

[35] Regulation (EU). 2017/745 of the European Parliament and of the Council of 5 April 2017 on medical devices, amending Directive 2001/83/EC, Regulation (EC) No 178/200 and 4. (2017).

