# Supplementary material for: Prevalence, attitudes and concerns toward telepsychiatry and mobile health self-management tools among patients with mental disorders during and after the COVID-19 pandemic: a nationwide survey in Poland from 2020 to 2023
Source: Front Psychiatry. 2024 Jan 8;14:1322695. doi: 10.3389/fpsyt.2023.1322695 (PMC10801431; doi:10.3389/fpsyt.2023.1322695)
Supplement: Supplementary file 1 [file Table_1.DOCX]

1 Supplementary Material – the full questionnaire

I. Prevalence and usage of new technologies

1) Do you use remote contact techniques with the specialist, i.e. video or teleconsultation?

a) Every day

b) Often (at least once a week)

c) Sometimes (at least once a month)

d) Rare (once a year or less often)

e) Never

2) Do you use internet-enabled mobile devices, i.e. a smartphone or tablet?

a) Every day

b) Often (at least once a week)

c) Sometimes (at least once a month)

d) Rare (once a year or less often)

e) Never

3) Do you know that there are mobile apps, smart watches, wristbands, etc. which support mental health monitoring and help manage mental disorders daily?

a) I know, I have recommended such solutions to patients

b) I know, I am interested in the topic

c) I know, but I am not interested

d) I have heard a little

e) I know nothing about it

II. Attitudes, expectations and preferences towards new technologies in psychiatry

1) Do you like the idea of using video/teleconsultation to contact a specialist?

a) Yes

b) I don’t know

c) No

2) Why?

3) Would you use video/teleconsultation if your doctor/psychologist recommended it?

a) Yes

b) No

4) In what situations would you like to use video/tele-consultation? (multiple-choice question)

a) First visit

b) As a complementary solution (alternating with traditional visits); As a continuation of treatment (subsequent visits)

c) Only in exceptional situations where no other contact is possible

d) Never

5) How often would you like to use video/teleconsultation to contact a specialist?

a) More than 70% of visits

b) 50-70% of visits

c) 30-50% of visits

d) less than 30% of visits

e) never

6) What would you like to improve in the current video/tele-consultation tools, do you see a need for new functionalities, if so which ones?

7) Do you like the idea of using mobile apps, smart watches, wristbands and other mobile health tools to support your care and treatment process?

a) Yes

b) I don’t know

c) No

8) Why?

9) What features in a mental health mobile app do you think would be useful for patients?

a) Educational - Psycho-educational materials to support management of the illness

i) Yes

ii) No

b) Self-motivating - Enabling the setting and achievement of goals

i) Yes

ii) No

c) Self-monitoring of well-being (e.g. sleep, mood, stress levels) with visualisation of this data e.g. in a chart and feedback

i) Yes

ii) No

d) Monitoring activity through e.g. call statistics, physical activity, mobility, voice parameters (to detect early signs of deterioration)

i) Yes

ii) No

e) Therapy support - Relaxation module

i) Yes

ii) No

f) Therapy support - Medication reminders

i) Yes

ii) No

g) Supporting therapy - Allowing on-going communication with the doctor/psychologist

i) Yes

ii) No

10) Other features that you think would be helpful?

11) Would you use a mental health mobile app if recommended by your doctor/psychologist?

a) Yes

b) I don’t know

c) No

12) How could using a mobile app with the sample features listed above affect your mental state?

a) Good

b) I don’t know

c) Bad

13) Assess your readiness to use new technologies

a) 1 (lack readiness)

b) 2

c) 3

d) 4

e) 5 (full readiness)

III. Concerns and risks associated with the use of new technologies in psychiatry

1) Do you have any concerns about the use of video/teleconsultations?

a) Yes

b) I don’t know

c) No

2) Which ones?

3) Do you have any concerns about the use of mobile apps and other mobile health tools to support care and treatment?

a) Yes

b) I don’t know

c) No

4) Which ones?

5) What other limitations/concerns do you see in the use of new technologies in psychiatry?

Metrics

1) Sex

a) Female

b) Male

2) Age

a) 18-24 years

b) 25-39 years

c) 40-55 years

d) 55-64 years

e) >65years

3) Education

a) Primary

b) Lower secondary

c) Professional

d) Higher

4) Professional activity

a) Student

b) Working

c) Pensioner

d) Other

5) Residence

a) Village

b) City up to 50,000

c) City 50,000-250,000

d) City >250,000

6) I am treated for… (multiple-choice question)

a) Depression

b) Anxiety

c) Sleep disorders

d) Addictions

e) Schizophrenia

f) Bipolar Disorder

g) Neurosis

h) Other

7) At present, I am…

a) Very seriously ill

b) Seriously ill

c) Noticeably ill

d) Moderately ill

e) Slightly ill

f) Almost health

g) Healthy
